# Community health workers and early detection of breast cancer in low-income and middle-income countries: a systematic scoping review of the literature

**DOI:** 10.1136/bmjgh-2020-002466

**Published:** 2020-05-13

**Authors:** James O'Donovan, Ashley Newcomb, MacKenzie Clark MacRae, Dorice Vieira, Chinelo Onyilofor, Ophira Ginsburg

**Affiliations:** 1 Department of Education, Oxford University, Oxford, Oxfordshire, UK; 2 Division of Research and Health Equity, Omni Med, Mukono, Uganda; 3 Perlmutter Cancer Center, NYU Langone Medical Center, New York, New York, USA; 4 Department of Medicine, Tufts University School of Medicine, Boston, Massachusetts, USA; 5 The George Washington University School of Medicine and Health Sciences, Washington, DC, USA

**Keywords:** cancer, health education and promotion, screening, systematic review, epidemiology

## Abstract

**Background:**

Breast cancer is the leading cause of female mortality in low-income and middle-income countries (LMICs). Early detection of breast cancer, either through screening or early diagnosis initiatives, led by community health workers (CHWs) has been proposed as a potential way to address the unjustly high mortality rates. We therefore document: (1) where and how CHWs are currently deployed in this role; (2) how CHWs are trained, including the content, duration and outcomes of training; and (3) the evidence on costs associated with deploying CHWs in breast cancer early detection.

**Methods:**

We conducted a systematic scoping review and searched eight major databases, as well as the grey literature. We included original studies focusing on the role of CHWs to assist in breast cancer early detection in a country defined as a LMIC according to the World Bank.

**Findings:**

16 eligible studies were identified. Several roles were identified for CHWs including awareness raising and community education (n=13); history taking (n=7); performing clinical breast examination (n=9); making onward referrals (n=7); and assisting in patient navigation and follow-up (n=4). Details surrounding training programmes were poorly reported and no studies provided a formal cost analysis.

**Conclusions:**

Despite the relative paucity of studies addressing the role of CHWs in breast cancer early detection, as well as the heterogeneity of existing studies, evidence suggests that CHWs can play a number of important roles in breast cancer early detection initiatives in LMICs. However, if they are to realise their full potential, they must be appropriately supported within the wider health system.

**Key questions**

**What is already known?**

- The incidence and mortality due to breast cancer are increasing in low-income and middle-income countries (LMICs) in various parts of the world.
- Early detection campaigns for breast cancer are not available for the majority of the world’s population, particularly those who live in LMICs.
- Community health workers (CHWs) have been suggested by the WHO to have a potential role in early detection of breast cancer in LMIC settings.

**What are the new findings?**

- This is the first study to systematically review the evidence across LMICs regarding the role of CHWs in the provision of breast cancer early detection services.
- From the 16 studies included in this review, CHWs appear to have a role in awareness raising and education; history taking and clinical breast examination; making onward referrals; and assisting individuals to navigate access to specialist care services, as well as conducting follow-up in the community.
- There are a relative lack of studies providing detailed descriptions of CHW training for breast cancer early detection, as well as the financial implications of such an approach.

**What do the new findings imply?**

- The use of CHWs to assist in breast cancer early detection in LMICs appears largely acceptable and feasible, although further studies evaluating the cost-effectiveness are warranted.
- Further studies evaluating the role of CHWs in breast cancer early detection initiatives within LMICs across geographically diverse populations with different genetic backgrounds, social values and lifestyles are warranted.

## Introduction

Breast cancer is the most common cancer in women globally^1 2^ and is the leading cause of cancer mortality in women in 103 countries worldwide.^3^ Over the past decade, the incidence of breast cancer in low-income and middle-income countries (LMICs) has increased significantly, and by 2020, it is estimated that 1.7 million new cases will occur in such countries.^3 4^ It has been estimated that of all total disability-adjusted life years lost due to breast cancer, almost 70% occur in LMICs.^5^ Furthermore, whereas survival rates from breast cancer in high-income countries (HICs) have been steadily improving over the past decade, those in LMICs have either stagnated or, in some cases, worsened.^6^

The reasons for these poor survival rates are complex and cut across ‘individual, interpersonal, organisational, community and policy issues’.^7–9^ The lack of early detection programmes in LMICs means that in many countries, as many as 75% of women with breast cancer present at an advanced stage (ie, clinical stage III or IV) resulting in worse outcomes.^10^ Second, even when breast cancers are detected early, treatment options are often limited, or otherwise less accessible, when compared with HICs.^4^ Third, competing disease burdens, lack of financing, political instability and a shortage of human resources^11^ mean breast cancer is not seen as a priority area in many LMICs.^12–14^ Therefore, given the burden of breast cancer in LMICs, there is a growing and pressing need to explore strategies by which to improve survival rates and quality of life.

One important way to improve survival rates from breast cancer is to improve early detection.^13 15^ The WHO defines early detection as ‘early diagnosis of people with symptoms, or screening of people without apparent symptoms’.^16^ In HICs, such as the UK, the National Health Service Breast Screening Program invites almost 3 million women aged between 50 and 64 years for screening every 3 years.^17^ This contrasts with the overwhelming majority of LMICs, where general population screening does not exist.^18^ However, a 2018 study by Birnbaum *et al*^6^ demonstrated that even without mammographic screening, improving the detection of breast cancer in LMIC settings where advanced disease presentation is common could be beneficial.^6^ Since mammography screening is not feasible in many LMIC settings, alternative low-cost screening approaches include breast self-examination (BSE) and clinical breast examination (CBE),^13 19^ which may be used in combination with advocacy and awareness campaigns to promote early detection of breast cancer.^20^

As a result, task shifting and up-skilling of lay health workers, commonly known as ‘community health workers’ (CHWs), has been proposed as one solution to address the shortage of individuals able to provide early detection services, especially in LMIC settings where there is a severe shortage of health workers’ impeding ability to achieve universal health coverage.^21^ The concept of training non-medical professionals to assist in the screening of breast cancer is not a new one,^22^ and the use of CHWs has helped to improve acceptability and increase uptake among minority and underserved groups of women in HICs such as the USA.^23–25^ Furthermore, although CHWs have been successfully trained and deployed to help reduce the burden of maternal and child health challenges, as well as infectious and other non-communicable diseases, their role to assist in breast cancer screening in LMICs is less clear. We identified only one systematic review by Wadler *et al*^26^ in 2011, which assessed the role of CHWs in South Africa to improve breast cancer control,^26^ and are unaware of any other reviews assessing the role of CHWs across LMICs more broadly.

As a result, this paper reviews the relevant literature from LMICs regarding breast cancer early detection to document:

- Where and how CHWs are currently deployed in this role.
- How CHWs are trained, including the content, duration and outcomes of training.
- The evidence on costs associated with deploying CHWs in early detection initiatives.

Based on the evidence from the literature, we will propose recommendations for the roles of CHWs in breast cancer early detection and outline priorities in early detection programmes more broadly.

## Methods

### Review approach

This systematic scoping review explores the role of CHWs in early detection of breast cancer in LMICs. The methods were modelled on a previous systematic scoping review published by the same lead author in 2018, which addressed the role of CHWs in cervical cancer screening in LMICs.^27^ The review process adheres to the Preferred Reporting Items for Systematic Reviews and Meta-Analyses (PRISMA).

A scoping review is ‘a form of knowledge synthesis that addresses an exploratory research question aimed at mapping key concepts, types of evidence, and gaps in research related to a defined area or field by systematically searching, selecting, and synthesizing existing knowledge’.^28^ Typically, they address broader research questions compared with a traditional systematic review, with less emphasis placed on critically appraising the included evidence.^29^

A review protocol was not published, and the study was not registered in the International Prospective Register of Systematic Reviews, in adherence with established guidelines for conducting scoping reviews.^28 30^ Nonetheless, we followed explicit and transparent research steps to explore the research evidence regarding early detection of breast cancer in LMICs by CHWs.

### Search strategy

With the assistance of a medical librarian trained in systematic review research (DV), the following eight databases were searched to identify primary, peer-reviewed studies published from 12 September 1978 up to and including 12 September 2019 on this topic:

- Biosis Citation Index.
- Embase.
- Food Science and Technology Abstracts (FSTA, by Ebsco).
- Global Health, by Ovid.
- PubMed/Medline.
- Scientific Electronic Library Online.
- Latin American and Caribbean Health Sciences Literature.
- Web of Science.

The strategy included subject headings and keywords for ‘Community Health Workers’, ‘Breast Cancer’, ‘Early Detection’ and ‘Low-Middle Income Countries’ (see online supplementary appendix S1 for full search details). Additionally, The New York Academy of Medicine Grey Literature and Online Computer Library Center’s OAISter databases were searched for additional scholarly information.

10.1136/bmjgh-2020-002466.supp1 Supplementary data

In order to capture all potentially relevant literature, we also searched the grey literature using the following sources: E-Theses Online Service, conference proceedings on Index of Conference proceedings and Google Scholar. Finally, we also searched the reference lists of all relevant papers that we identified, using snowball sampling.

### Inclusion and exclusion criteria

The inclusion criteria were:

1. The primary focus of the study must be breast cancer early detection (ie, screening or early diagnosis).
2. CHWs must be the primary participants of the study.
3. The study must take place in a LMIC, as defined by World Bank classifications, at the time of data collection.

Studies were excluded if:

1. They were reviews, narratives, commentaries or abstracts.
2. They primarily focused on the role of doctors, nurses, students, allied health workers or allied healthcare workers, that is, health professionals other than CHWs.
3. They were not specifically related to breast cancer early detection; for example, they focused primarily on cervical cancer screening and mentioned breast cancer screening in passing with insufficient detail for data analysis.

#### Population

For this study, we used the widely accepted WHO definition of CHWs, which is: ‘Community health workers should be members of the communities where they work, should be selected by the communities, should be answerable to the communities for their activities, should be supported by the health system but not necessarily a part of its organization, and have shorter training than professional workers’.^31^

#### Intervention

Studies had to focus on the role of CHWs in the early detection of breast cancer. According to the WHO, early detection can be defined either as screening of an asymptomatic population or early diagnosis of individual displaying symptoms.^16^

#### Comparator

A comparator was not included.

#### Outcomes

The outcomes for our review were documenting the geographical location of existing studies where CHWs had a role in breast cancer early detection, the roles they played, the methods used to train and support CHWs and associated outcomes following training and the financial costs associated with deploying CHWs to facilitate with early detection.

### Study selection

Papers identified during the initial search were exported into the reference management software EndNote 7.1. Duplicate references were removed through a combination of automated and manual deduplication. Titles and abstracts of all publications identified in the search were screened by three authors (AN, CO and MCM) to determine whether they would be considered for a full-text review using Covidence. A fourth reviewer (JO) reviewed screener conflicts and broke all ties.

Studies that were clearly not about breast cancer early detection were discarded at this stage. Following this initial screening process, the full text of the remaining studies was reviewed against the inclusion and exclusion criteria by the same four authors (JO, AN, CO and MCM). This was done independently by each author before a joint discussion was held to break ties.

### Data analysis

Once studies were determined to have met the inclusion criteria, data were systematically extracted from each study and tabulated using a data charting form in an online shared Microsoft Excel spreadsheet. The use of a data charting form has been recommended by Arksey and O’Malley^29^ and Levac *et al*^30 32^ as a key stage of conducting a scoping review.^30 32^ Data extraction variables included: World Bank region and LMIC tier, study purpose, design and setting, early detection strategies (further divided into ‘screening’ or ‘early diagnosis’), names, roles and descriptions of CHW cadres, description of CHW training and assessment, results of economic evaluation or cost analyses and study results and impact (see online supplementary appendix S3 for full data extraction table).

### Patient and public involvement

Patients and the public were not involved in the design or execution of this study.

## Results

### Search results

**Figure 1:**
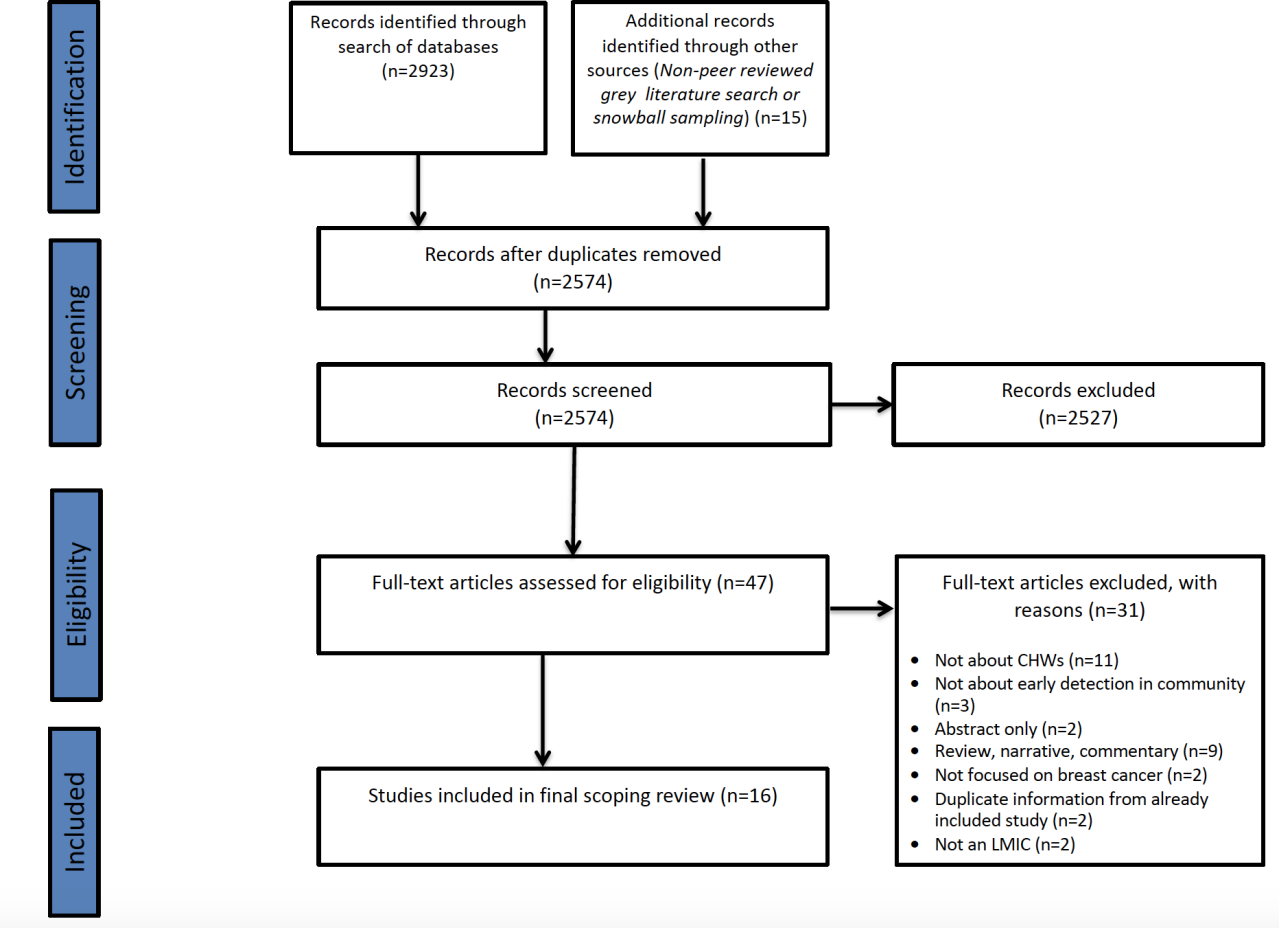
PRISMA diagram. A PRISMA diagram outlining the search strategy and study filtering process. LMIC, low-income and middle-income country; PRISMA, Preferred Reporting Items for Systematic Reviews and Meta-Analyses.

The initial search of eight databases, the grey literature and snowball sampling yielded 2938 results. After deduplication, 2574 studies were screened via their title and abstract. Following this initial screening process, the full texts of 47 studies were obtained for a full-text review. Following this full-text review against the inclusion and exclusion criteria, 31 studies were excluded. Reasons outlining the exclusion of studies at this stage can be found in the PRISMA flow chart (figure 1).

On completion of the full-text review, 16 studies remained for inclusion in the study,^2 33–45^ of which one originated from the search of the grey literature.^37^ It is important to note that two sets of studies included in this review were linked. First, the study by Chowdhury *et al*^2^ was a pilot feasibility study for the larger study published by Ginsburg *et al*.^2 34^ Second, the study by Kohler *et al*^33^ was a qualitative exploratory study of the larger project reported by Gutnik *et al*^36 38^

### Where do CHWs currently have a role in breast cancer early detection?

**Figure 2:**
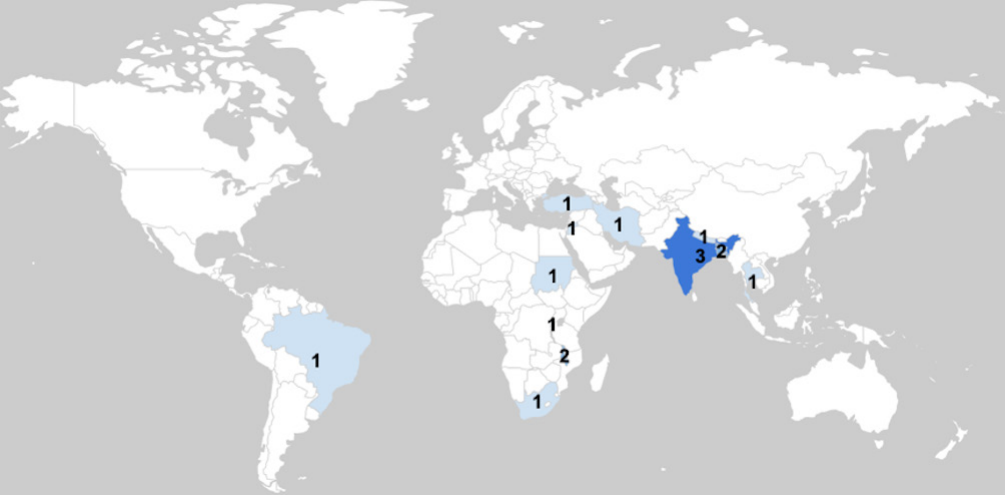
Choropleth map. A choropleth map highlighting the location of studies included in this review.

The 16 studies included in this review took place between 2005 and 2019, in 12 countries, representing five World Bank regions: South Asia (n=6),^2 34 37 39 43 46^ sub-Saharan Africa (n=5),^33 36 38 42 45^ Middle East and North Africa (n=3),^35 41 44^ East Asia and the Pacific (n=1)^47^ and Latin America and the Caribbean (n=1)^40^ (see figure 2, choropleth map). At the time of data collection, the studies took place both in rural (n=10)^2 33 34 37 40 42 43 45–47^ and urban settings (n=6),^18 35 38 39 41 44^ as well as in countries with economic statuses ranging from low income (n=5),^34 36 38 42 46^ to lower middle income (n=7)^2 33 37 39 41 43 47^ and upper middle income (n=4).^35 40 44 45^

### CHW descriptions

A total of 12 different terms were used to describe CHWs and the number of CHWs involved in each study ranged from 4 CHWs^36^ to 1 076 CHWs^42^; however, in four studies, this was not documented.^37 39 40 47^

The descriptions provided for CHW cadres also varied significantly across the studies. Five studies provided no description regarding the regular daily roles and duties of the CHWs (n=5),^37 38 41 43 47^ and in seven studies, CHWs were specifically recruited for the purpose of that particular study (n=7).^2 33–36 38 45^ Where descriptions were provided, they commonly constituted a basic demographic description of the CHWs (n=11). For example, the description given to the CHWs involved in a study in Malawi by Gutnik *et al*^36^ was ‘laywomen recruited from the local community who were paid to take on the role of Breast Health Worker’.^36^ Importantly, 63% (n=10/16) of studies had solely female CHWs.^2 33–36 38 39 43 44 46^

Only one study from Rwanda, by Pace *et al*,^42^ provided a detailed description regarding the characteristics of the CHWs, as well as their responsibilities beyond breast health.^42^ In this study, CHWs (62% of who were female) were described as playing several roles, including building community awareness about preventive healthcare and connecting individuals to the wider healthcare system. The mean age of CHWs in this study was 40 years, and the majority had a primary school education (76.9%). Furthermore, 54.6% of CHWs had been practising for 6–10 years at the time of the intervention. Only 5.5% had previous training in breast health, and only 51.1% had heard of breast cancer before, with only 9.1% of the group having held community awareness raising sessions before this.

### How are CHWs currently deployed in breast cancer early detection initiatives in different studies?

Of the 16 studies that described a role for CHWs in early detection of breast cancer, 13 described CHWs involvement in screening programmes of asymptomatic women^2 33 35–41 43 45–47^ and 3 described CHWs roles in early diagnosis of symptomatic women.^2 34 44^ One study did not specifically describe a role for CHWs in a screening or early diagnosis programme but rather provided an in-depth assessment of a training programme designed for CHWs to assist in early detection more generally and was thus deemed appropriate for inclusion in the final review.^42^ In the studies where early diagnosis was selected over general population screening, methods including ‘case finding’ were deemed more appropriate given the low incidences of breast cancer, young age of onset and difficulty in follow-up from initial presentation. In all 16 studies, CHWs had a variety of different roles in early detection initiatives. These included:

#### Awareness raising and education

The most common role for CHWs described across 14 studies was in awareness raising and education.^2 33–40 42–45 47^ Activities included CHWs conducting door-to-door home outreach visits in the community to raise awareness about breast cancer,^33 35 40 45^ providing educational talks at community health centres and communal areas (such as cafés and places of worship),^33 36 38^ handing out pamphlets and information leaflets,^37^ showing motivational videos on mobile phones^2 34^ and teaching women how to perform their own BSE.^39 43 44 47^

For example, in the linked studies by Gutnik *et al*^36^ and Kohler *et al*,^38^ four CHWs delivered educational talks in the waiting rooms at local clinics to both men and women. Over a 4-month period in 2015, ‘2860 women and 1435 men attended 175 talks in five clinics’.^36^ In subsequent interviews, women who had attended the talks managed to retain knowledge opposing commonly held false-beliefs, such as holding money in a bra does not cause cancer, and reported sharing information with peers who had not attended the talks. Women also found the pictures on the flip charts used during the talks to be particularly useful, with one stating: ‘it was explaining well even for a person who doesn’t know how to read, you can just point on the flipchart and know what cancer is’.^38^

In studies by Kamproh and Fungpong, Kulkarni *et al*, Rao *et al* and Taha *et al*,^39 43 44 47^ women were taught by CHWs on how to conduct BSE, which appeared to have positive effects on the frequency on which BSE was conducted. For example, in the study by Taha *et al*^44^ after being taught BSE, the number of women reporting regular self-examination increased from 27% at baseline to 96% after 6 months (n=593).^44^

#### History taking and data collection

In seven studies, CHWs took histories from women to collect sociodemographic data, elicit symptoms of breast cancer and explore potential risk factors.^2 33–36 38 44^ For example, in Turkey, 30 CHWs were trained to use a structured form to collect data from 5100 women such as their sociodemographic details and risk factors for breast cancer,^35^ and in Bangladesh, 30 CHWs were trained in how to use mobile phones to collect similar data.^34^

In the study by Gözüm *et al*,^35^ 5100 women in Turkey were reached by CHWs. The intervention appeared to have a positive effect on BSE rates since it was noted that prior to the CHW intervention, 66.4% of women did not perform a regular BSE, compared with just 38.1% after 8 months.^35^

#### Performing CBEs

In nine studies, CHWs were trained to carry out CBE.^2 33 34 36 38 39 41 42 46^ For example, Abuidris *et al*^33^ found that a door-to-door screening programme of 10 309 women in Sudan by 29 CHWs conducting CBEs helped increase the detection of breast cancer in asymptomatic women residing in rural communities.^33^ Similarly, Kulkarni *et al*^39^ demonstrated that CHWs conducting CBE ensured good compliance with screening, referral and treatment, indicating acceptability and feasibility within the community.^39^ Gutnik *et al*^36^ found that CBE done by CHWs compared well with physician exams, yielding ‘a sensitivity of 94% (95% CI 79% to 99%), specificity of 58% (95% CI 46% to 70%), positive predictive value of 48% (95% CI 35% to 62%), and negative predictive value of 96% (95% CI 85% to 100%)’.^36^

#### Onward referrals

Seven studies described how CHWs made referrals for onward specialist review and further investigation.^35–38 40 46^ For example, in the study by Abuidris *et al*^33^ in Sudan, out of the 10 309 women screened, 138 were referred to the National Cancer Institute at Gezira University by the CHW with a suspected abnormality.^33^ Although 118 women attended for further assessment, of whom nine were later diagnosed with breast cancer, 20 women did not attend the follow-up. Although this study did not explicitly explore the reasons as to why this happened, one hypothesis was that no financial assistance was given to facilitate transport. In other studies where women did not attend referrals, reasons given included a lack of time, household duties and fear of a diagnosis of breast cancer.

#### Patient navigation and follow-up

Four studies described a role for CHWs in patient navigation and follow-up at various stages of the early detection cycle.^2 34 39 44^ For example, Ginsburg *et al*^34^ assessed the efficacy of CHWs trained in patient navigation to improve treatment adherence throughout the cancer care pathway in India.^34^ This study was a three-arm cluster controlled trial, where group A was CHWs provided with a smartphone containing an app for data collection, in addition to a motivational video and the ability to offer an appointment to women with abnormal CBEs; group B was CHWs also with a smartphone, but in addition offered patient navigation; and group C was the control group with no smartphone and no patient navigation service but collected data using traditional paper forms. The study found that the women assigned to a CHW in arm B (*smartphones plus patient navigation*) were significantly more likely to attend for care versus women in arm A (smartphones without navigation; 63% vs 43%, p=0.0001).

Similarly, in the study from India by Kulkarni *et al*,^39^ CHWs counselled women prescreening and were also based at the health centre in order to assist women through the diagnostic work-up stage.^39^ Importantly, in this study, women were provided with transport to the hospital appointments, thus helping to reduce barriers to access.

### How are CHWs trained and assessed for early detection of breast cancer?

Fourteen of the 16 included studies provided some description of how training and assessment of CHWs was conducted; however, there was significant heterogeneity across studies in terms of details provided, as well as how training programmes were designed, delivered and evaluated (see online supplementary appendix S3).

The majority of CHW training programmes were described as 5 days or less in duration (n=8),^2 33–35 41–44^ while Gutnik *et al*^36^ conducted training over a 4-week period^36^ and Tum *et al*^45^ conducted training over 3 months.^36 45^

Content of training programmes also varied depending on the aims of the study. For example, the study by Taha *et al*^44^ provided a description of a comprehensive and holistic training course where CHWs were taught by a certified local female trainer over a 3-day period.^44^ Course content ranged from lectures about local breast cancer statistics and national guidelines to practical sessions focusing on communication skills, and breast examination technique, as well as group work aimed at addressing common myths and cultural barriers. In other programmes, such as that by Kamproh *et al*,^47^ training focused on one specific element, such as BSE.^47^

Training styles, faculty and materials also varied. Several studies reported the involvement of multiple stakeholders, including ministry of health officials, breast cancer survivors, nurses and oncologists,^33 36 47^ whereas others were led by one stakeholder, such as a nurse.^45^ Trainings ranged from information dissemination models such as lectures^46^ to interactive workshops with practical demonstrations using model breasts,^41^ role-play exercises and case discussions.^36^ The heterogeneity across these categories is perhaps reflective of the variability of locally available resources.

Evaluation of CHW training was generally poorly documented across studies, with seven studies providing no details regarding how CHWs were assessed. Where evaluation was documented, CHWs were mainly assessed through tests or examinations, such as multiple-choice tests,^35^ preknowledge assessments and postknowledge assessments^36^ and written tests,^42 45^ or in one case through observed competencies in clinical breast exams.^33^ Hyoju *et al*^46^ used interobserver agreement rates to evaluate the clinical skill transfer from surgeons to CHWs.^46^

### What are the financial considerations for deploying CHWs in breast cancer early detection?

No study conducted a formal economic evaluation regarding the use of CHWs in the early detection of breast cancer, although four studies stated whether CHWs were financially compensated for their time. In the studies by Ginsburg *et al*,^34^ Gutnik *et al*^36^ and Tum *et al*,^45^ it was noted that CHWs were given a small financial stipend to compensate them for their time; however, exact cost figures were not provided.^34 36 45^ Conversely, Abuidris *et al*^33^ and Gozum *et al*^35^ noted that the CHWs in their respective studies were not paid, which they hypothesised could represent a potential barrier to long-term sustainability.^33 35^

### Challenges with deploying CHWs to facilitate early detection

Although the response and impact of deploying CHWs to assist in the early detection of breast cancer was generally positive, there were also challenges noted across the studies. For example, in the study in Sudan, 6 of the 35 villages that initially agreed to participate in the project did not send a volunteer to the initial CHW training session, meaning some CHWs then had to serve villages other than their own.^33^ Of note, women in two of these villages refused to be seen by a non-resident CHW. There was also individual variability in screening coverage across the 35 villages, which correlated with the participation of community leaders in the project. Those villages whose community leaders engaged with the project had higher rates of screening than those which did not. Local stakeholder buy-in and engagement therefore appears critical to the uptake of such services. Rates of screening were also highly variable at the individual village level; in some villages no women were screened, whereas in others there was a 100% screening rate. It therefore has to be highlighted that there can be large variability among individual CHWs in terms of motivation and activity within the community.

Other challenges noted across different studies concerned the accuracy of data collection and entry by the CHWs. For example, although none of the 2029 women who were interviewed in the study by Chowdhury *et al*^2^ voiced concern over their data being collected on mobile phones, it was subsequently noted that 20% (n=405) of case entries contained data errors surrounding patient and household identification information.^2^

## Discussion

This scoping review highlights the diverse roles CHWs have in the early detection of breast cancer across 12 LMICs. These roles include awareness raising and education; history taking and CBE; making onward referrals; and assisting individuals to navigate access to specialist care services, as well as conducting follow-up in the community. The reported training of CHWs was highly variable, and no studies conducted a formal cost analysis. Although generally positive, the impact of using CHWs to facilitate in the early detection of breast cancer was not without its challenges, ranging from examples of reluctance towards being screened by non-native CHWs to accuracy of data recording.

Given the burden of breast cancer morbidity and mortality in LMICs, the role of CHWs in early detection initiatives is relatively underexplored, with only 16 studies identified in this review. Furthermore, since the majority of studies (69%; n=11/16) took place in either sub-Saharan Africa or South Asia, more studies across other regions are urgently needed, given the contextual variability regarding disease burden, cultural norms and tertiary care infrastructure. These context-specific studies can also help to inform policy makers in individual countries as to which early detection approach might be more suitable, depending on factors such as incidence rates, age at onset, workforce and resource availability, as well as sociocultural and structural barriers that might be an impediment to a general screening programme, compared with targeted early diagnosis of high-risk populations. Importantly, only three studies reported early diagnosis initiatives, whereas the WHO and the Breast Health Global Initiative recommend that for resource-limited settings priority should be given to scaling up capacity to manage clinically palpable disease first and supporting initiatives to increase/improve access to early diagnosis. We therefore wish to caveat the following discussion by highlighting that general population screening should not be considered relevant until health systems are able to support it.^48 49^ We also wish to caveat that the specific modality (CBE, BSE and mammography) used in early detection and screening of breast cancer in LMICS is highly contested.^13 19 20^ We therefore present recommendations based on the synthesis of existing evidence, rather than making recommendations on the effectiveness of screening modalities.

### A proposed model for CHWs in the early detection of breast cancer

**Figure 3:**
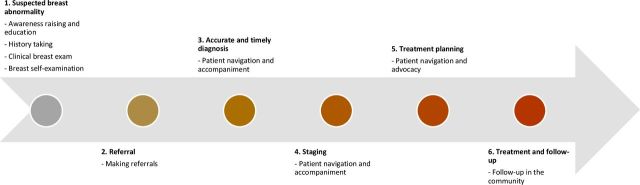
Roles for CHWs at each stage in a patient's breast cancer journey.

Breast cancer detection, diagnosis and treatment involve a complex pathway with multiple stages—from the initial point of referral following a potential abnormality to timely diagnosis and staging, treatment planning and access and through to follow-up. From the studies identified in this review, CHWs have been documented to have several key roles at each of these stages. We therefore mapped the existing evidence surrounding CHWs’ current involvement in the early detection of breast cancer and beyond over the course of a patient journey (figure 3).

At the most basic level, CHWs should have a role in breast cancer awareness raising and community education. Based on the studies included in this review, this could take different forms depending on availability of local resources but could include distribution of information leaflets, sharing of motivational videos, holding education sessions and door-to-door home visits. For maximum impact, we recommend that awareness-raising activities are tailored to the context in which they are delivered and that during the design phase, provision is made to reach the most marginalised populations, such as those in rural and remote areas. Given that CHWs are from the communities in which they work, are often selected due to good community standing and have shared commonalities with the target population (such as language and sociocultural characteristics), they represent ideal individuals to fulfil this role.

A second important role for CHWs is in history taking. CHWs could combine community education and awareness raising with enquiring about symptoms related to breast cancer and counselling women with concerning symptoms to seek care. It is important to note, however, that supervision for this role will be imperative given that in the study by Chowdhury *et al*,^2^ several inaccuracies in data input were noted.^2^ It is also important to note that community members should be fully aware as to how their data will be used and that with the proliferation of mobile technologies to collect data, data security must be optimised. CHWs should also be trained in how to ask questions in a sensitive matter, given the stigma attached to breast cancer in many LMICs.^50^

A third role for CHWs during the initial early detection stage is conducting CBEs. In LMIC settings, CBE is one low-cost and feasible approach towards the early detection of breast cancer,^51^ especially given the relative lack of imaging modalities commonly deployed in HIC settings for screening, such as mammography. Beyond lack of access to imaging modalities, the lack of radiographers to perform and interpret mammograms is also problematic.^51^ Furthermore, there is an ongoing discourse questioning the usefulness of mammography for population-level screening, given potential overdiagnosis and a minimal effect on reducing mortality from women presenting with advanced breast cancer.^52^ Although CBE has not been recommended as a primary screening modality due to the limited evidence in LMIC settings, there is emerging evidence that women presenting with a new diagnosis of breast cancer are more likely to be diagnosed at an earlier stage of disease if they report having had a previous CBE (unrelated to current diagnosis).^53 54^ It has also been included as an option in the resource-tiered guidelines of the 2010 Breast Health Global Initiative Consensus,^48^ which suggests that in countries with limited resources, positive CBE could be supplemented with imaging modalities such as diagnostic breast ultrasound (±diagnostic mammography), with the aim of downsizing symptomatic disease.^52^ Finally, in addition to performing CBE, CHWs should teach women how to conduct BSE. While BSE has not been shown to be an effective primary screening method, it is considered an important aspect of breast health education (sometimes called ‘breast awareness’).^55^

For stages beyond the initial detection of a suspicious breast lesion, CHWs can have an important role in referral, advocacy and subsequent patient navigation. Given the complexities of referral systems and subsequent follow-up, this represents an important role for CHWs beyond initial early detection. Existing studies in LMIC settings have demonstrated that women with breast cancer typically have to pass through multiple points of care before they reach the appropriate referral hospital, and at each step, there is a risk of dropout.^56^ In other contexts, such as the USA, CHWs have been successfully deployed since the 1990s as patient navigators to help underserved groups navigate the formal health system.^57^ Such roles can be important in helping to ‘close the loop’.

Taking a ‘high level perspective’, underpinning a CHW early-detection model is the requirement for general programmatic considerations. This includes appropriate CHW selection, optimising training and ongoing support mechanisms and strengthening specialist oncology services in LMIC settings more broadly.

Selection of CHWs for early detection should be grounded in context-specific needs and accepted practices. For example, in some societies female CHWs may be strongly preferred to men for the purposes of conducting CBE; indeed the qualitative study by Kohler *et al*^38^ from Malawi found that 30% of women stated that they would feel uncomfortable with a male CHW performing a CBE.^38^ Similarly, training programmes should be culturally appropriate and tailored to the setting for which they are being designed for, and assessments should move beyond simplistic pretest and post-test assessments of knowledge, towards real-world assessments of CHWs daily practice, which could incorporate communication and clinical skills. Trainings should also target men as well as women. The study by Gutnik *et al*^36^ was an excellent example of how educational talks were delivered at primary health clinics, where both men and women were in attendance.^36^ Such initiatives could help encourage open conversations around cancer and break down stigmas attached to the disease. It is also important that future studies provide more detail on training design, content and outcomes, so that best practices can be developed and shared across contexts. It is also an important caveat that six of the studies included in this review did not consider the role of CHWs in the early detection of breast cancer in addition to their other roles and responsibilities. It is therefore unclear as to how such approaches would fare if they were added to the already high workload of CHWs, as well as how the additive effect would impact CHW motivation, performance and quality of services being provided.

Furthermore, it will be important that those responsible for the design and administration of early detection initiatives consider how marginalised individuals within LMICs benefit, given existing in-country disparities. For example, individuals living in rural areas experience higher levels of mortality due to women being diagnosed in the later stages of disease, which are less responsive to treatment.^58^ CHWs being deployed in rural areas could be one way to help close this gap, as well as bringing staging services closer to the community. This was highlighted in the study by Mauad *et al*^40^ by conducting mammograms in a mobile van equipped with mammography equipment.^40^ Such initiatives can help reduce some of the barriers faced by women in rural areas when trying to access specialist staging services, such as a lack of transport and long distances to specialist hospitals. Similarly, in the study by Chowdhury *et al*,^2^ over half of five-person households reported an income of less than $3/day.^2^ A recent study from Vietnam by Hoang *et al*^59^ estimated that 37.4% of households would be driven to financial catastrophe if they were to meet current treatment costs if one member of their household were to be diagnosed with cancer.^59^ Bringing diagnostic services closer to the community, and having dedicated CHWs accompany patients to follow-up appointments, might help to reduce some of these costs to the individual.

Finally, there is a pressing need for a greater number of studies exploring the financial implications of deploying CHWs to assist in early detection initiatives. Many CHWs involved in the studies covered by this review were women, who were not recompensed for their labour.^60–62^ This mirrors findings from other contexts, such as Nepal, where already financially poor female health volunteers often make out-of-pocket payments to deliver maternal health services.^63^ Future studies exploring the role of CHWs in breast cancer early detection should make provision for financial recompense of CHWs to ensure that they are be paid for their work, in keeping with WHO guidelines for optimising CHW programmes.^64^

### Limitations

The quality of evidence included in this review was not assessed, meaning we are therefore unable to make recommendations based on the quality of evidence; however, this is in keeping with established guidelines for conducting systematic scoping reviews. There will also be ongoing initiatives involving CHWs in breast cancer early detection that have not been captured by this review if they have not been formally published. The heterogeneity of the studies also made it challenging to draw conclusions across studies given that outcome measures assessing the impact of CHWs on breast cancer screening were variable.

## Conclusion

CHWs can have an important role to play in early detection of breast cancer in LMICs, with responsibilities including awareness raising, conducting CBEs, making referrals and supporting subsequent patient navigation. However, this promise can only be turned into genuine progress if they are appropriately supported. This will involve adopting contextually appropriate early detection initiatives that are embedded within the broader health system, where CHWs are appropriately trained, equipped, paid and supported with appropriate links to specialist oncology services. Above all, early detection programmes in LMICs must make provision for *every* individual at risk of breast cancer—this will mean considering the needs of the hardest to reach first, so that no woman is left behind in the goal to end unjust and untimely deaths from the leading cause of female mortality in LMICs.
