# Supplementary material for: Community health workers and early detection of breast cancer in low-income and middle-income countries: a systematic scoping review of the literature
Source: BMJ Glob Health. 2020 May 13;5(5):e002466. doi: 10.1136/bmjgh-2020-002466 (PMC7228495; doi:10.1136/bmjgh-2020-002466)
Supplement: Supplementary data [file bmjgh-2020-002466supp001.pdf]

SUPPLEMENTARY MATERIAL

S1. Full list of search terms. A full list of search terms used to search each database.

A. Community Health Workers

(accompagnateurs OR activista OR Agente comunitario de salud OR Agente comunitario de saude OR Agent de Sante Communautaire OR Agentes Polivalentes Elementares OR Allied health personnel OR anganwadi OR Animatrice de Sante Maternelle OR Accredited Social Health Activist OR ASHA OR Auxiliary health worker OR Barangay health worker OR Barefoot doctor OR Basic health worker OR behvarz OR binome OR brigadista OR Care Facilitator OR Colaborador voluntario OR Community based reproductive health agents OR Community assistant OR Community care worker OR Community development worker OR Community drug distributor OR Community health representative OR Community health advocate OR Community health agent OR Community health aide OR Community health educator OR community health assistant OR Community health educator OR Community health promoter OR Community health volunteer OR Community health worker OR Community health extension worker OR Community nutrition worker OR Community mobilizer OR Community resource person OR Community reproductive health worker OR Community support worker OR Community volunteer OR Community-based worker OR Female community health volunteer OR Female multipurpose health worker OR Frontline health worker OR Health extension worker OR Health and nutrition worker OR Health service assistant OR Health surveillance assistant OR Health promoter OR Home health aide OR Health Volunteer OR Home visitor OR kader OR Lady health worker OR Lay health visitor OR Lay health worker OR Link worker OR Maternal and child health promotion worker OR Maternal and child health worker OR Mental health worker OR monitira OR Mother coordinator OR Outreach educator OR Paramedical worker OR Patent Medicine Vendor OR Peer volunteer OR Postnatal support worker OR Primary health care worker OR promotora OR raedat OR Relais communautaire OR Rural health motivator OR Rural health worker OR Rural health care worker OR Saksham sahaya OR sevika OR Shastho karmis OR Shastho shebika OR visitadora OR Village drug-kit manager OR Village Health Guide OR Village health helper OR Village health worker OR Village health committee OR Village health team OR Volunteer health worker OR Voluntary Community Health Worker OR Women group leader)

B. Breast Cancer

(breast neoplasms OR breast neoplasm OR breast cancer OR breast tumor OR breast tumors OR breast carcinoma OR mammary cancer OR mammary carcinoma)

C. Early Detection

(screening OR clinical breast exam OR clinical breast examination OR CBE OR cancer screening OR early detection OR early diagnosis OR cancer screening test OR self-examination OR detection OR early detection of cancer)

D. Low-and Middle-Income Countries

(developing countries or developing country or medically underserved area or medically underserved areas or medically underserved areas LMIC or low income countries or low income country or middle income countries or middle income country or global or resource poor or low resource or third world country or third world countries or Africa or Central Asia or Western Asia or Southeastern Asia or Indian Ocean Islands or Central America or South America or Eastern Europe or Transcaucasia or China or Korea or Mongolia or Mexico or Caribbean Region or Pacific Islands or Africa or Sub-Saharan Africa or Sub Saharan Africa or Africa South of the Sahara or Central Asia or Western Asia or Southeastern Asia or Indian Ocean Islands or Central America or South America or Eastern Europe or Transcaucasia or Caribbean or Pacific Islands or Afghan or Afghani or Afghanistan or Bangladesh or Bangladeshi or Benin or Beninese or Burkina Faso or Burkinabe or Burundi or Burundian or Cambodia or Cambodian or Central African Republic or Central African or Central Africa or Chad or Chadian or Comoros or Comoran or Congo or Congolese or Eritrea or Eritrean or Ethiopia or Ethiopian or Gambia or Gambian or Guinea or Guinean or Haiti or Haitian or Kenya or Kenyan or Korea or Korean or Kyrgyz or Kyrgyzstan or Liberia or Liberian or Madagascar or Malagasy or Malawi or Malawian or Mali or Malian or Mozambique or Mozambican or Myanmar or Myanmarese or Burmese or Nepal or Nepalese or Niger or Nigerien or Nigeria or Nigerian or Rwanda or Rwandan or Sierra Leone or Sierra Leonean or Somalia or Somalian or Tajikistan or Tajik or Tadzhik or Tanzania or Tanzanian or Togo or Togolese or Uganda or Ugandan or Zimbabwe or Zimbabwean or Angola or Angolan or Armenia or Armenian or Belize or Belizean or Bhutan or Bhutanese or Bolivia or Bolivian or Cameroon or Cameroonian or Cape Verde or Cape Verdian or Cape Verdean or Cote d'Ivoire or Ivory Coast or Ivorian or Djibouti or Egypt or Egyptian or El Salvador or Salvadorian or Salvadorans or Fiji or Fijian or Georgia or Georgian or Ghana or Ghanaian or Guatemala or Guatemalan or Guyana or Guyanese or Honduras or Honduran or Indonesia or Indonesian or India or Indian or Iraq or Iraqi or Kiribati or Kosovo or Kosovar or Laos or Lao or Laotian or Lesotho or Marshall Islands or Marshallese or Mauritania or Mauritanian or Micronesia or Micronesian or Moldova or Moldovan or Mongolia or Mongolian or Morocco or Moroccan or Nicaragua or Nicaraguan or Nigeria or Pakistan or Pakistani or Papua New Guinea or Papua New Guinean or Paraguay or Paraguayan or Philippines or Filipino or Samoa or Samoan or Sao Tome or Principe or Santomean or Senegal or Senegalese or Solomon Islands or Solomon Islander or Sri Lanka or Sri Lankan or Sudan or Sudanese or Swazi or Swaziland or Syria or Syrian or East Timor or East Timorese or Tonga or Tongan or Turkmenistan or Turkmen or Tuvalu or Tuvaluan or Ukraine or Ukrainian or Uzbekistan or Uzbek or Vanuatu or Vietnam or Vietnamese or West Bank or Gaza or Yemen or Yemeni or Yemenite or Zambia or Zambian or Albania or Argentinian or Azerbaijan or Azerbaijani or Belarus or Belarusian or Bosnia or Bosnian or Botswana or Brazil or Brazilian or Bulgaria or Bulgarian or Barbados or Bajan or Barbadians or Chile or Chilean or China or Chinese or Colombia or Columbian or Costa Rica or Costa Rican or Dominica or Dominican or Ecuador or Ecuadorean or Gabon of Gabonese or Grenada or Grenadian or Iran or Iranian or Jamaica or Jamaican or Jordan or Jordanian or Kazakhstan or Kazakhstani or Latvia or Latvian or Lebanon or Lebanese or Libya or Libyan or Lithuania or Lithuanian or Macedonia or Macedonian or Malaysia or Malaysian or Maldives or Maldivian or Mauritius or Mauritian or Mauritania or

Mauritanian or Mexico or Mexican or Montenegro or Montenegrin or Namibia or Namibian or Palau or Palauan or Panama or Panamanian or Peru or Peruvian or Romania or Romanian or Russia or Russian or Serbia or Serbian or Seychelles or Seychellois or South Africa or South African or Saint Kitts or Saint Lucia or Saint Vincent or Suriname or Surinamer or Thailand or Thai or Tunisian or Turkey or Uruguay or Venezuela or Venezuelan or Guinea Bissau or Western Africa)

S2 Table. Database search results. A table outlining the number of results generated from each individual database (after de-duplication).

**Database**	**Number of hits**
Biosis	34
Embase	207
Journal of Food Science and Technology Abstracts (FSTA, by Ebsco)	3
Global Health, by Ovid	202
PubMed/Medline	1027
Scientific Electronic Library Online (SciELO)/ Latin American and Caribbean Health Sciences Literature (LILACS)	8
Web of Science +	1093

S3 Table. Summary table of included studies

https://doi.org/10.7910/DVN/LDRYO2
